# Mucosal washes are useful for sampling intestinal mucus-associated microbiota despite low biomass

**DOI:** 10.1080/19490976.2025.2464296

**Published:** 2025-02-20

**Authors:** Jennifer N. Martinez-Medina, Fereshteh Ghazisaeedi, Catharina Kramer, Jörn F Ziegler, Victoria McParland, Paul W. Mönch, Britta Siegmund, Víctor Hugo Jarquín-Díaz, Marcus Fulde, Sofia K. Forslund-Startceva

**Affiliations:** a Max-Delbrück-Center for Molecular Medicine in the Helmholtz Association (MDC), Berlin, Germany; b Charité – Universitätsmedizin Berlin, Corporate Member of Freie Universität Berlin and Humboldt-Universität zu Berlin, Berlin, Germany; c Experimental and Clinical Research Center, a Cooperation of Charité-Universitätsmedizin and The Max-Delbrück Center, Berlin, Germany; d Institute of Microbiology and Epizootics, School of Veterinary Medicine at the Freie Universität Berlin, Berlin, Germany; e Veterinary Centre for Resistance Research (TZR), School of Veterinary Medicine at the Freie Universität Berlin, Berlin, Germany; f Department of Gastroenterology, Infectious Diseases and Rheumatology, Berlin, Germany; g Berlin Institute of Health at Charité – Universitätsmedizin Berlin, BIH Biomedical Innovation Academy, BIH Charité Junior Clinician Scientist Program, Berlin, Germany; h DZHK (German Centre for Cardiovascular Research), Berlin, Germany; i Structural and Computational Biology, European Molecular Biology Laboratory, Heidelberg, Germany

**Keywords:** Mucus, intestinal microbiota, mucus-associated, low-biomass, mice, humans, mucosal-washes, biopsy, brushes, bacterial load

## Abstract

Understanding the dynamic relationship between mucus-associated microbiota and host health is critical. However, studies predominantly using stool samples may not accurately represent these bacterial communities. Here, we investigated the mucus-associated microbiota in the gastrointestinal tract of mice and the terminal ileum of humans using different sample types: mucosal washes, brushes, scrapings, and intestinal contents in mice and biopsies, brushes and mucosal washes in humans. We used DNA quantification and 16S rRNA amplicon sequencing to evaluate the comparability of the information yielded from the different sample types under a controlled benchmark. In mice, mucosal washes and brushes had comparative bacterial DNA and host DNA contamination than scraping samples. Similarly, in humans, washes outperformed biopsies in bacterial DNA content. Read counts and microbiota alpha diversity remained remarkably similar in mice and between brushes and washes in humans. The composition of the microbiota varied based on the subsegment and sample type in mice and sample type in humans. We conclude that washes and brushes reduce host contamination without inducing substantial compositional bias when sampling mucosal microbiota. Our findings suggest that mucosal washes and brushes are a viable alternative to biopsies in humans and scrapings in mice, thereby improving the transferability of results across hosts. Our study highlights the importance of focusing on mucus-associated microbiota to better capture host–microbiome interactions at their closer interface.

## Introduction

Assessing the taxonomic composition and functional profiles of the intestinal microbiome provides relevant insights into the mechanisms and determinants of host health and disease.^1–3^ The well-being of the host depends in part on a balanced interaction with the microbiota throughout life and development, just as the microbiota depends on its host. In general, disruption of this balance promotes the development of chronic diseases, such as inflammatory bowel disease.^4^ Most studies to date have focused on the fecal microbiota, which is only imperfectly correlated in either role or composition with the microbial communities found in the intestinal mucus, or the directly mucus-associated microbiota, which have a much closer physiological relationship to the host.^5^

The mucus is a glycoprotein-based secreted by the goblet cells that forms a layer that covers the intestinal epithelium and provides a barrier between the lumen and the underlying tissue.^6^ The properties and composition of this mucus are essential for the establishment and maintenance of the associated microbiota.^7–9^ Proper characterization of the microbial communities inhabiting the mucus layer relies on the use of an appropriate sampling methodology that is representative of the biology under study.

In the mouse models commonly used to study intestinal diseases, the samples selected to study the mucus-associated microbiota are mainly obtained from the luminal contents, by scraping or washing the mucosal surface, and from whole tissues at dissection. In contrast, in humans, the most commonly used sample types for the same purpose are biopsies, mucosal–luminal interface aspirates, colonic lavages, and endoscopic brushings.^10–13^ Mucosal biopsies are considered to be the main sample used to characterize human mucus-associated microbiota.^10^ However, they have several disadvantages, including: 1) a reduced representation of the sampled intestinal subsegment and 2) a high content of host DNA, which can interfere with the bacterial DNA signal and further interpretation of its results, especially as the mucosal interface itself, in contrast to the luminal content, is a sample with low bacterial biomass.^10,14^ In addition, it is important to identify a protocol that works well under both clinical and preclinical conditions so that results from these modalities remain methodologically comparable yet reflect the limitations of these settings, at the sampling of patients as well as the size of small model organisms such as mice.

Body sites and samples with low bacterial biomass pose a major challenge for the study of mucus-associated microbial communities. Several attempts have been made to improve the assessment of mucus-associated microbiota. One approach is to deplete host DNA from biopsies by using different extraction methods to enrich bacterial DNA prior to sequencing.^14^ Other alternatives involve different sample types, such as lavages, for reproducible substitution of mucosal biopsies.^10,11^ Furthermore, the variation in the sample type used within even the same host could pose technical and biological challenges for the interpretation of the results and the comparison between studies. Nevertheless, there is no consensus on the best choice of sampling method for the assessment of mucus-associated microbiota in human or animal models.^5,10,15^

A critical step to better characterize the mucus-associated microbiota is to evaluate the reliability of low biomass samples such as biopsies, scrapings, or mucosal washes and compare them to luminal content from the small and large intestines of different host origins. Such benchmarking is necessary to assess the differences between samples obtained by different methods and between samples from different anatomical subsegments. This may help to clarify the advantages and limitations of the respective techniques and provide a basis for possible large-scale study implementations. Here, we aim to compare the mucus-associated microbiota in different sample types collected from three gastrointestinal subsegments in mice, and from the terminal ileum in humans. We are doing this to identify a protocol that will allow clinical-preclinical comparisons and improved reproducibility. This is to gain a more robust and translatable understanding of the health implications of variation in the intestinal mucus-associated microbiome.

## Materials and methods

### Study sample

All animal experiments were approved by the local office of occupational health and technical safety “Landesamt für Gesundheit und Soziales, Berlin” LaGeSo Reg. Nr. T 0284/15. Animals were maintained in the Institute of Microbiology and Epizootics, School of Veterinary Medicine at the Freie Universität Berlin, Robert-von-Ostertag-Str. 7 14,163 Berlin, Germany. We collected samples from C57BL/6J-congenic Toll-like receptor 5 (TLR5) null mice (B6(Cg)Tlr5<tm1.2Gewr>/J − 028909) (Tlr5-d) (*N* = 6) and wild-type (WT) (*N* = 6) male mice, each of 8 weeks of age per group. These mice were under a maintenance diet for this study. The knock-out genotype was selected as previous work showed it to exhibit phenotypes where alterations to the mucosal microbiome are expected.^16^

Human samples were obtained at the Department of Gastroenterology, Infectious Diseases and Rheumatology, Charité - Universitätsmedizin Berlin, Berlin, Germany, between 2021 and 2024, and approval from the ethical committee of Charité Universitätsmedizin Berlin (EA4/120/20) was obtained. Samples were collected from 13 controls (12 mucosal washes, 6 biopsies and 10 brushes) and 24 Crohn’s disease (CD) patients (24 mucosal washes, 4 biopsies and 9 brushes). Biopsies, mucosal washes, and brushings were taken as part of an already scheduled colonoscopy and written informed consent was obtained. For the control group, indications for the exploratory colonoscopy were symptoms like abdominal pain, diarrhea, weight loss, surveillance, or endometriosis. For patients with CD, colonoscopy was performed to assess disease activity, surveillance, or specific symptoms. Patients in all stages of the disease were included (before or under treatment, and remission), while patients were excluded if they had undergone an ileocecal resection or an increased procedural risk in colonoscopy, like due to cardiopulmonary comorbidities or to the intake of anticoagulants. Although we included patients with CD, this study was not designed to detect differences between healthy or diseased groups. A larger sample size would be required to achieve statistical power for epidemiological conclusions, and the present study only aims to define the sampling methodology for mucus-associated microbiota in patient-derived samples.

### Collection of low-biomass sample types and intestinal content samples

**Figure 1.**
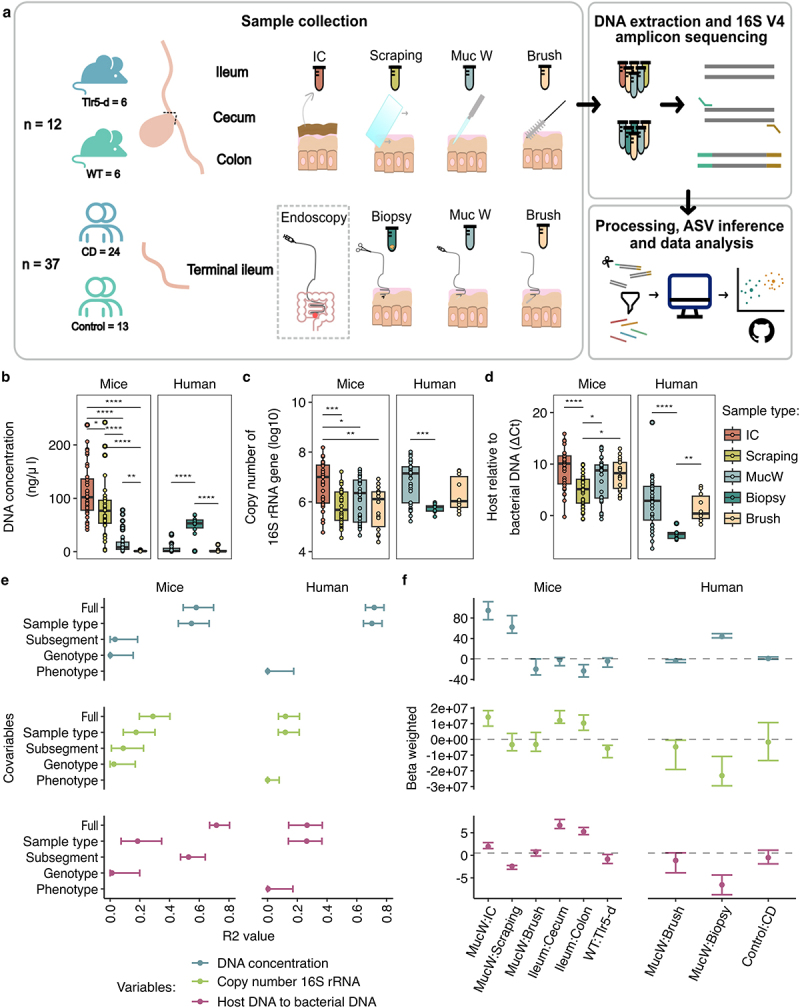
General assessment of bacterial DNA associated with mucus between sample types in mice and humans. a) Methodology of the study, sample collection including mice and human samples taken with different protocols. In mice and humans, b) the DNA concentration (ng/uL), c) the bacterial load, assessed as 16S rRNA gene copy number, and d) abundance of host DNA relative to bacteria DNA assessed by the δct method. The higher the δct value, the higher the amount of bacteria DNA in the sample. Each point represents an individual sample. e) Variance explained (R^2^) in a forest plot of the DNA concentration, the bacterial load and the host relative to bacterial DNA (δct) in mice (left) and humans (right), with the 95% confidence interval for each fixed effect in the models with marginal R^2^values. f) Beta estimation of the models per host for DNA concentration (ng/uL), bacterial load and, host DNA relative to bacteria DNA (δct). Margins of the forest plots represent 95% confidence intervals. CD: Crohn’s disease; IC: Intestinal content; MucW: Mucosal washes.

We collected mucosal washes, brushes, scraping samples from the ileum, cecum, and colon of mice. The intestinal contents from the same subsegments were also collected as a reference for the luminal microbiome. After removing the intestines, we isolated the three subsegments of interest and opened them longitudinally. The ileum was defined as the final third of the small intestine, while the colon corresponded to the first third of the large intestine following the cecum. For each mouse, three or four tissue pieces were collected per segment (ileum, cecum, and colon) to obtain the different sample types (Figure 1(a)). The intestinal contents per subsegment were mixed with ~1 mL of phosphate buffered saline (PBS), removed, and collected. Mucosal washes were collected by gently injecting ~1 mL PBS into the lumen of each subsegment and recovering the solution. Scraping samples were collected by sliding microscope cover slips across the luminal surface of tissue subsegments with the mucus, transferring the sample into a collection tube containing ~1 mL PBS. Mucosal brushes were obtained after removing the intestinal content. Cytobrushes (B9 1200) were gently pressed and rolled on the luminal surfaces and the brushes were then transferred to tubes containing 1 mL of PBS. All samples were collected in 2 mL tubes, snapped frozen in liquid nitrogen and stored at −80°C until further processing.

Human terminal ileum mucosal washes were obtained by colonoscopy using a sterile catheter 230 cm long and 2.3 mm wide (Endo-Flex GmbH). A sterile 10 mL syringe (PosiFlush^TM^ SD) was used to flush physiological saline solution (NaCl 0.9% w/v) onto the mucosal surface in the terminal ileum of the patient. Approximately 4–6 mL of the resulting intestinal fluid was collected. Biopsies of about 2 × 2 mm were obtained from the same subsegment using forceps per usual procedural practice. Mucosal brushes were collected by colonoscopy using a sterile RX Cytology Brush (2.1 mm brush x 8F (2.7 mm)). Upon reaching the terminal ileum, the brush was extracted from its cover. Gentle pressure was applied to brush the mucosa, ensuring comprehensive contact with all sides of the brush. After retracting the brush into its cover, the instrument was withdrawn from the endoscope. Then, the extracted brush was cut off and placed into 0.9% saline solution. All samples were snap frozen in liquid nitrogen and stored in microcentrifuge tubes at −80°C.

### DNA extraction

As negative controls, we included water molecular biology grade to be processed alongside all extractions and NaCl 0.9% solution used to collect human samples. All samples and negative controls were extracted using ZymoBIOMICS^TM^ DNA Miniprep Kit (ZYMO Research Europe GmbH, Freiburg, Germany).^17^ The starting material among available for the intestinal contents, mucosal washes, and scraping samples from mice ranged between 24 and 500 mg, 260 and 500 μl, and 13 and 240 mg, respectively. The amounts used for human mucosal washes and whole biopsies ranged from 250 to 600 μl and 3 to 28 mg, respectively.

All samples and controls were mixed with 750 μL of ZymoBIOMICS Lysis Solution, followed by a Proteinase K incubation step using a preheated ThermoBlock at 55°C for 30 min. Bead-beating homogenization was done only for biopsies using a PeQLab Precellys 24 (Bertin Corp., Rockville, MD, USA) for 2 × 15 s at 5500 rpm with beads of 2.0 mm (ZR BashingBead, Lysis Tubes of ZymoBIOMICS). The next steps followed the manufacturer’s protocol. DNA was eluted with 50 μL of DNase/RNase Free Water in a 1.5 mL microcentrifuge tube by centrifuging at 16,000 × g for 3 min. Samples were stored at −80°C until further experiments. DNA quantification and the quality check was performed using spectrophotometry in a NanoDrop (PEQLAB Biotechnologie GmbH, ND-1000) and Qubit dsDNA BR Assay Kit or dsDNA HS Assay Kit (Invitrogen by Thermo Fisher Scientific).

### Absolute quantification of bacterial load and relative quantification of host DNA

Absolute quantification of the bacterial load was done for all sample types and extraction controls. All amplifications were performed in triplicates in 96-well optical plates (Applied Biosystems) with a final volume of 10 μL containing 5 μL of a 2× SYBR Green PCR Master Mix including a passive reference dye (Applied Biosystems), 10 μM of each primer (Univ337 F 5′-ACT CCT ACG GGA GGC AGC AGT-3′ - Univ 518 R 5′-GTA TTA CCG CGG CTG CTG GCA C-3′ or Univ218 F 5’-ACT GAG ACA CGG CCC A-3’ - Univ515 R: 5’-TTA CCG CGG CMG CTG GCA C-3’) and 1 μL of template DNA (2.5 ng/µL final concentration). Copy numbers per ng of DNA were estimated based on a standard curve prepared with the amplification of the 16S rRNA gene of *E. coli* (27F: 5’-GTT TGA TCC TGG CTC AG-3’ and 1492 R: 5’-CGG CTA CCT TGT TAC GAC-3’, by Invitrogen, C404010) in dilutions from 10^3^ to 10^9^. A standard amplification protocol of Applied Biosystems QuantStudio3 AppliedBiosystem (Thermo Fisher Scientific, Darmstadt, Germany) was followed: For samples of mice and humans, amplification was made with 95°C for 20 s followed by 40 cycles at 95°C for 3 s and 60°C per 30 s. In all cases, a melting curve (Tm) analysis was performed, increasing the temperature from 60°C to 95°C at a rate of 0.2°C per second with the continuous monitoring of fluorescence to check for specificity.

Relative quantification of mouse DNA was made using primers (Ms_gDNA_CDC42_F: 5’-CTC TCC TCC CCT CTG TCT TG-3’ and Ms_gDNA_CDC42_R: 5’-TCC TTT TGG GTT GAG TTT CC-3’) for *Mus musculus* nuclear single copy gene *Cdc42*,^18^ and SYBR-Green (Applied Biosystems) PCR Master Mix and molecular biology grade water. Amplification conditions for all DNA from mice samples were a denaturation step at 95°C for 2 min, 40 cycles at 95°C for 15 s and 55°C per 15 s. To determine the human host DNA content, we amplified a region of the 18S rRNA gene using the primer pairs F: 5’-ACA TCC AAG GAA GGC AGC AG-3’ and R: 5’-TTT TCG TCA CTA CCT CCC CG-3’, amplification conditions were a 95°C for 20 s, 40 cycles at 95°C for 3 s and 60°C per 30 s, with a final melting step.

### Microbial community pre-processing

Amplification of the 16S rRNA gene in the V3-V4 regions was made with Klindworth primers pair (341F: 5’-CCT ACG GGN GGC WGC AG-3’ and 785 R: 5’-GAC TAC HVG GGT ATC TAA KCC-3’) from DNA extracted of all the different sample types. Library preparation and sequences were generated by LGC Genomics (LGC Genomics GmbH, Berlin) on the Illumina MiSeq platform in two runs using “v3 chemistry” with 600 cycles (2 × 300bp). All sequencing raw data can be accessed through the BioProject: PRJNA1043131 in the NCBI Short Read Archive (SRA).

Sequencing reads were processed following filtering by quality check, amplicon sequence variants (ASVs) were inferred and taxonomically assigned using the package DADA2 v1.18.0.^19^ Sequences were trimmed to two different conditions based on the host of origin. For sequences from mice samples, we used the setting truncLen = c(280,210), while for sequences from human samples we used setting truncLen = c(280,230). In both cases, we allowed a maximum error of 2 nucleotides and removed the Phix spike-in. Forward and reverse sequencing reads were de-replicated, concatenated and chimeras were removed. Taxonomic assignment was done using the SILVA database (v138.1)^20^ with the RDP naive Bayesian classifier.^21^

The final dataset contained only ASVs assigned at least at the family level, and we removed ASVs classified as mitochondrial or chloroplast. All taxonomic data, ASV abundance table and metadata were compiled into a single object using the package Phyloseq v1.34.0.^22^ A decontamination processing was done using the package Decontam v1.10.0.^23^ We used the combine-either methodology to identify possible bacteria at the genus level classified as contaminants based on their frequency and prevalence with the thresholds (0.1, 0.4, respectively) for mice and human data. Additionally, we included literature research to define the final contaminants, based on whether taxa previously were reported as contaminants or not. Samples with less than 100 reads were filtered out. The read counts were rarefied and transformed to relative abundance for the differential abundance analysis of ASVs at phylum and genus levels.

### Diversity estimation

Beta diversity assessment is based on the Aitchison distance of raw counts, based on Euclidean distances, using the centered log-ratio (CLR) transformation on the data matrix to avoid issues of compositionality.^24^ We applied constrained Principal Component Analysis (PCA) to ordinate objects based on such intra-sample distances and visualize the multidimensional data, using the vegan R library.^25^ Alpha diversity after rarefaction was evaluated using the Chao1 index for richness taxa estimation, the Shannon index that depends on the richness and evenness of the taxa, and the Inverse Simpson metric, which focuses on a weighted mean of proportional abundances.^26^

### Statistical analysis

Normality was evaluated with the Shapiro test in qualitative variables (DNA concentration, copy number 16S, delta host DNA) using the function shapiro_test() in the R package rstatix (v.0.7.1),^27^ and non-parametric statistics were used when a normal distribution could not be concluded. Comparisons of copy number, host DNA quantity, alpha diversity and differential abundance at different levels of the taxonomy between samples of different types were made using the Kruskal–Wallis test, followed by Dunn’s post hoc test to evaluate per pair of groups in mice and human samples, and with Mann–Whitney U test in paired-human samples to evaluate the comparisons between two different sample types from the same patient. All multiple comparisons were corrected by the False Discovery Rate (FDR) with the Benjamini–Hochberg procedure.^28^ The functions used were kruskal_test(), dunn_test() and wilcox_test(), respectively, in the rstatix library.^27^

For beta diversity, redundancy analysis (RDA) was used to model the effects of our different covariates on the entirely microbial community. The test was run on the CLR abundance of ASV tables using as explanatory variables’ sample type, subsegment (only in mice), genotype or phenotype and controlling per individual. A PCA was applied to the fitted matrix to reduce the dimensionality and obtained the eigenvalues that were compared throughout ANOVA analysis with 999 permutations using ANOVA function with vegan package in R. Generalized Linear Mixed Models (GLMMs) were run with the lme4 package function lmer().^29^ To determine the partition of the variance explained in the complex GLMM we used the partR2 package with the function partR2().^30^ The output of this analysis reveals the variance denoted by R^2^ and the direction and size of the effect indicated by the beta coefficients. The response variables included quantitative variables such as bacterial load, alpha diversity indexes and read counts, and the predictors included the sample types or the genotype or phenotype groups, paired for contrast taking one category as reference. The plots were created with ggplot2().^31^ All the scripts for the analysis described here are available at https://git.bihealth.org/ag-forslund/hydrogels_sampletype.

## Results

We collected intestinal contents from mice in addition to four types of mucus samples, including mucosal washes and brushes from both hosts, scrapings from mice, and biopsies from humans (Supplementary Table S1). In a total of 12 mice, sampling included the collection of three samples (intestinal content, mucosal washes, and scrapings), and in 6 out of 12 mice a collection of a fourth type of sample (mucosal brushes) was taken. Each sample was collected from three different gastrointestinal subsegments per individual. For the human cohort, we included controls (*n* = 13) and patients with CD (*n* = 24), for a total of 10 biopsies, 36 mucosal washes and 10 mucosal brushes taken from the terminal ileum (Figure 1a; Supplementary Table S1).

### Intestinal mucosal wash sampling captures an equivalent and comparative bacterial load as scraping, mucosal brushes or biopsy sampling

To determine whether mucosal washes could capture equivalent amounts of bacteria as sampling by scrapings, mucosal brushes, or biopsy, we quantified and observed the resulting DNA concentration and the bacterial load between sample types. In both host species, DNA concentration and bacterial load were significantly different between the sample types (Figure 1(a); Supplementary Table S2). Mucosal wash samples exhibited lower DNA concentrations than scrapings or intestinal content samples but the mucosal brush samples in mice and biopsies in humans (Figure 1(b)).

Despite lower overall DNA concentrations, mucosal washes and brushes had higher values of bacterial content than scrapings or biopsy samples (Figure 1(c), Supplementary Table S2). Similar results were observed for paired human mucosal washes-biopsies (*n* = 9), but no differences were observed between the paired human mucosal washes-brushes (*n* = 10; Supplementary Figure S1a-b; Supplementary Table S3). Additionally, in mice, we compared the bacterial load between the sample types in three subsegments: the ileum, cecum and colon. The bacterial load was similar between mucosal washes, brushes and scrapings in the ileum. In the colon and cecum, the bacterial load of the mucosal washes compared to scrapings, and it was slightly higher than brushes. The latter did not reach significance between the sample types, but the intestinal content. Overall, the intestinal content was higher in all subsegments (Supplementary Figure S1b).

To estimate the abundance of host DNA, we used a relative quantification of host DNA genes compared to the bacterial 16S rRNA gene. For mouse samples, we used a *Mus musculus* single-copy nuclear gene *Cdc42*, and for human samples, a region of the 18S rRNA gene. We used ΔCt between host and bacteria as a proxy for host DNA. Thus, the higher the ΔCt value, the higher the content of bacterial DNA in the samples. Mucosal washes and brushes yielded significantly less host DNA than scrapings and biopsy samples in mice and humans, respectively (Figure 1(d)). This was also consistent within subsegments, with statistically differences in colon and cecum between mucosal washes and scrapings, and in colon and ileum for brushes (Supplementary Figure S1c).

To determine the effect of biological covariates (genotype or phenotype, subsegment) on the three variables of interest, we used individual generalized linear mixed models (GLMMs) with DNA concentration, bacterial load and host DNA as our response variables. For mice, the GLMMs included mouse identification number (ID) and the batch as random effects and subsegments, genotypes (WT or Tlr5-d), and sample type (scrapings, mucosal washes, brushes, or intestinal contents) as fixed effects. For humans, we included patient ID and batch as a random effect with phenotype (control or CD) and mucosal washes, brushes, and biopsy as fixed effects.

We observed that sample type explained most of the variation for the total DNA and the bacterial load in both mice (R^2^_marginal_  = 0.548; R^2^_marginal_  = 0.175) and humans (R^2^_marginal_  = 0.702; R^2^_marginal_  = 0.119). For the relative evaluation of host DNA to bacterial DNA (ΔCt), the differences were mostly described by the subsegment in mice (R^2^_marginal_  = 0.528) and the sample type in human samples (R^2^_marginal_  = 0.263) (Figure 1(e)). In all cases, the genotype or phenotype contributed less than 1% of the variation in mice and humans, respectively (Figure 1(d); Supplementary Table S4).

The beta estimation of the model was used to determine the direction of the effect. The intestinal content in mice showed higher total DNA, bacterial load and higher relative host to bacterial DNA contrasted to the mucosal washes. The brush and scraping samples showed a similar result for the bacterial load, with higher DNA for scrapings and lower for brushes compared to mucosal washes (Figure 1(f)). In human samples, the biopsy had a higher DNA concentration, while mucosal brushes were lower in contrast to mucosal washes (Figure 1(f)). However, brushes and biopsies yielded lower bacterial loads in humans. On the Tlr5-d mice, we observed a slightly lower size effect of bacterial load compared to WT mice and with the same effect in the phenotype between all controls and CD patients on DNA concentration, bacterial load, or host DNA (Figure 1(f)).

Furthermore, statistical pairwise contrasts of the models of the bacterial load between the sample types within the three subsegments were statistically different mainly for intestinal content in comparison to mucosal washes, brushes, and scraping sample types, with a higher bacterial load for intestinal content (Supplementary Table S5). For human samples, differences in bacterial load were observed when contrasting mucosal washes against biopsy sample types within the same phenotype group (Supplementary Table S5).

The results suggest that intestinal mucosal washes and brushes contain less host DNA than scrapings or biopsies, making them a preferable method for reducing the loss of bacterial resolution due to host DNA contamination in sequencing. While bacterial load is comparable between mucosal washes and scrapings in mice, it remains lower in biopsies compared to mucosal washes in humans.

### Mucosal washes, scrapings, and brushes read counts are similar for the evaluation of the mucus-associated microbiota

To understand the impact of sample type on sequencing results, we quantified its effect on total reads as well as reads remaining after taxonomy filtering and decontamination using the Decontam v1.10.0 package.^23^ We identified a total of 6 and 15 specific contaminants in mice and humans, respectively (Supplementary Figures S2a-f). Of these, one mouse contaminant (*Cutibacterium*) and two human contaminants (*Delftia, Sphingomonas*) were found in the extraction controls. We excluded ASVs previously classified as contaminants by the Decontam library but known to inhabit the host intestine, otherwise they were discarded as contaminants (Supplementary Table S6). ASVs classified as *Cutibacterium* were also excluded from human samples as they had a prevalence of 17%.

**Figure 2.**
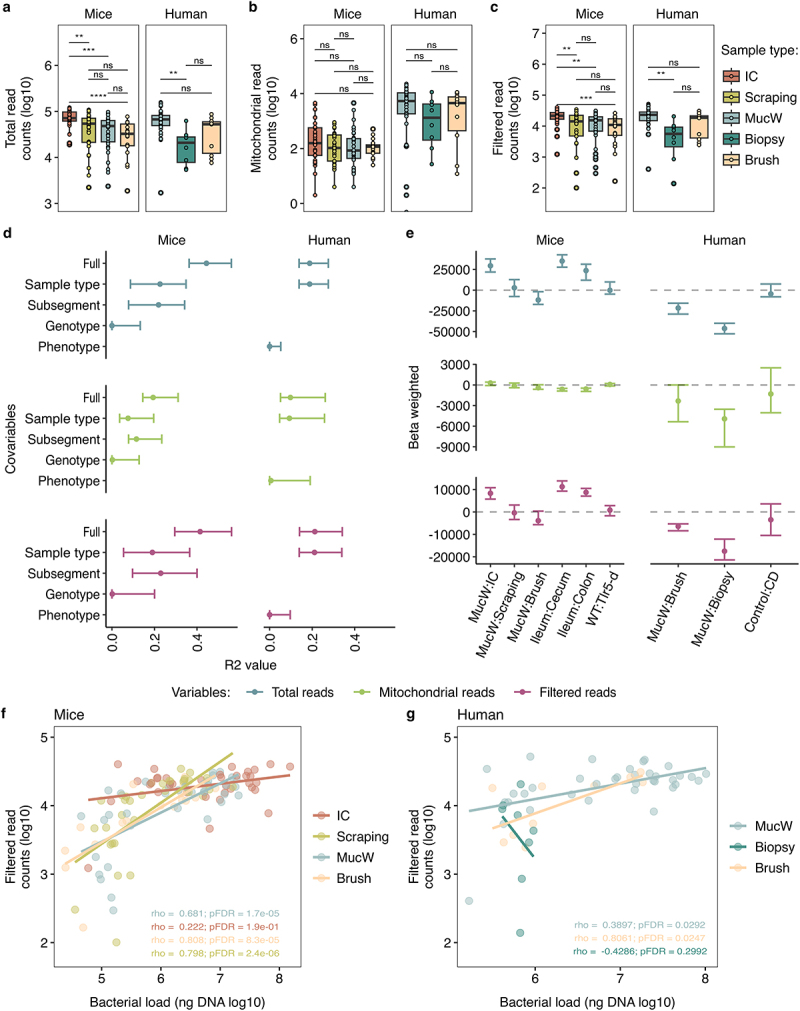
Assessment of sequencing reads between sample types in mice and humans. a) Total read counts, b) mitochondrial reads, c) filtered read counts, represented in logarithm 10. d) Variance explained (R^2^) in a forest plot of the total, mitochondrial and filtered reads in mice (left) and humans (right), with the 95% confidence interval for each fixed effect in the models with marginal R^2^values. e) Beta estimation of the models per host mice (left) and human (right). Margins of the forest plots represent 95% confidence intervals. f-g) Spearman correlation between the bacterial load (ng of DNA represented in logarithm 10) and the filtered read counts (represented in logarithm 10) in f) mice and g) humans. IC: Intestinal content; MucW: Mucosal washes.

The number of total mitochondrial-associated and decontaminated bacterial reads was similar in mucosal wash samples compared to scrapings and brush samples in mice, in the all set of samples or on the results per subsegment (Figure 2(a–c); Supplementary Figure S3a-c). Conversely, in humans, mucosal washes tended to higher values for all the read variables (Figure 2(a–c); Supplementary Figure S3a-c). The evaluation on the statistical model with the independent variable (sample types) and the remaining covariates showed that the variance for the total reads was explained mainly by the sample type (R^2^_marginal_ = 0.226), the filtered and mitochondrial reads by the subsegment (R^2^_marginal_ = 0.229 and R^2^_marginal_ = 0.115, respectively). On the human data, the total, filtered and mitochondrial read variables were mostly explained by the sample type (R^2^_marginal_ = 0.188, R^2^_marginal_ = 0.211, R^2^_marginal_ = 0.093, respectively) (Figure 2d and Supplementary Table S4). We also observed that mucosal wash samples generally yielded fewer total or filtered reads than intestinal content samples in mice, and similar results were observed between the other sample types or for the mitochondrial reads (Figure 2(e)). Interestingly, in humans, the total or filtered reads had a lower effect from biopsies and mucosal brushes in contrast to mucosal washes. For mitochondrial reads, the brushes did not differ in content contrasting to mucosal washes but were lower for the biopsies. On both phenotypes, a similar effect was observed in the three read variables (Figure 2(e) and Supplementary Table S4).

Pairwise contrast of the effect sizes of the total and filtered reads reached significance in mice when comparing the different low biomass samples (scrapings, mucosal washes and brushes) against the intestinal content, but no differences were observed between the contrast test within the low biomass samples within the subsegments or the genotype (Supplementary Table S5). Human samples had a significant difference in total and filtered reads when comparing the mucosal washes against biopsies or brushes stratifying within CD patients or control patients (Supplementary Table S5).

We evaluated whether the bacterial load was associated with the decontaminated bacterial reads in the samples. We performed a Spearman correlation between the two proxies. Higher bacterial load was strongly correlated with higher decontaminated bacterial reads in mucosal washes, brushes and scraping samples from mice (rho >0.6, pFDR <0.01). Comparatively, the mucosal washes and brushes had a positive correlation between the bacterial load and the decontaminated bacterial reads in human samples (rho >0.2, pFDR < 0.05) (Figure 2(f–g)). These results showed that the mucosal washes and brushes are likely to have comparative reads to assess the mucus-associated microbiota in mice and humans.

### Diversity and composition of the microbiota varies per intestinal subsegment while the mucosal washes and the brushes could give similar information

**Figure 3.**
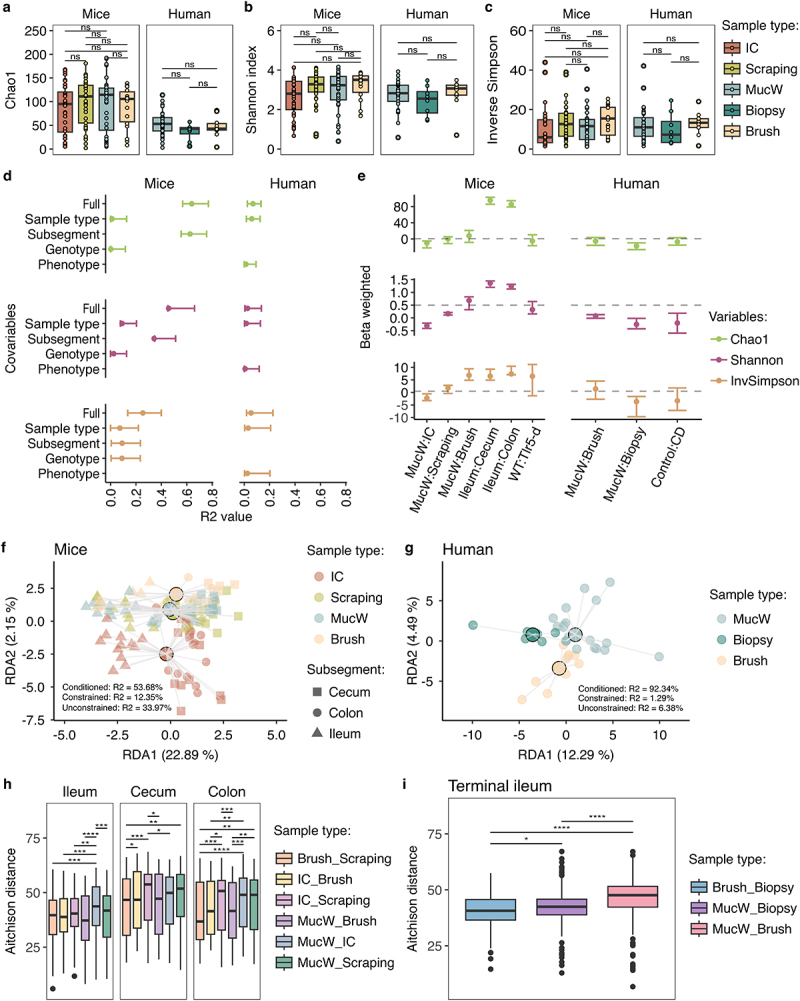
Assessment of alpha and beta diversity between sample types in mice and humans. Alpha diversity presented with the metrics: a) Chao1, b) Shannon, c) inverse Simpson (InvSimpson) in mice (left) and humans (right). Ns: not significant. d) Variance explained (R^2^) in a forest plot of the alpha diversity metrics, with the 95% confidence interval for each fixed effect in the models with marginal R^2^values in mice (left) and humans (right). e) Beta estimation of the models per host mice (left) and human (right). Margins of the forest plots represent 95% confidence intervals. f-g) Principal Component Analysis (PCA) using Redundancy analysis (RDA) of the beta diversity represented with Aitchison distance in f) mice and g) humans. Points with a black stroke line represent the centroids of the sample types. h-i) Comparison between paired sample types of the Aitchison distances (similarity between the sample types), in h) mice samples were represented stratified per subsegment, and i) in humans they were represented by the total of sample per sample type in the terminal ileum. IC: Intestinal content; MucW: Mucosal washes. *p < 0.05, **p < 0.0, ***p < 0.001, ****p < 0.0001.

To assess intra-individual microbial diversity, we calculated the Chao1 index for ASV richness estimation, Shannon, and Inverse Simpson indices. Notably, we did not observe statistically significant differences between sample types in either mouse or human samples, suggesting comparable diversity readouts from the different sample types (Figure 3(a–c); Supplementary Figure S4a-c).

We applied GLMM models to investigate the factors influencing alpha diversity metrics and to observe the effect size in mice and humans. Alpha diversity metrics are essential for assessing species diversity within a community. For mice, our analysis revealed that the primary factors influencing the variation in Chao1, Shannon and evenness (inverse Simpson) diversity were related to the subsegment (Chao1: R^2^_marginal_  = 0.626; Shannon: R^2^_marginal_  = 0.343; Inverse Simpson: R^2^_marginal_  = 0.091) (Figure 3d; Supplementary Table S4). A lower effect on the three alpha diversity indices was observed for the mucosal washes compared to the intestinal content, while a lower Shannon diversity was observed for the scrapings and a higher evenness for the brushes compared to the mucosal washes (Figure 3(e); Supplementary Table S4). The higher diversity in the lumen of the intestinal content compared to the mucus-focused washes was similar for the scraped mucus and the brushes.

In the human dataset, the primary driver of variation in alpha diversity metrics was the sample type (Chao1: R^2^_marginal_  = 0.0596; Shannon: R^2^_marginal_  = 0.017; Inverse Simpson: R^2^_marginal_  = 0.032) (Figure 3(d); Supplementary Table S4). The biopsies had a smaller effect size on the three alpha diversity indices, while the brushes had a lower Shannon diversity compared to the mucosal washes (Figure 3(e); Supplementary Table S4). This means that in humans, the mucus washes could add more information about diversity at the mucus level than the biopsies.

Our pairwise comparisons within the classification per subsegment on the output of the generalized mixed model on Chao1 did not show significant differences between the sample types, but for Shannon, differences were observed between the intestinal content and mucosal washes, brushes or scrapings on the three subsegments, while for inverse Simpson, the difference was between the brushes versus the mucosal wash and the intestine content (Supplementary Table S5). No differences were observed in humans (Supplementary Table S5). These results provide insight into the key factors that may influence alpha diversity in mice and humans, as the effect of subsegment predominated in mice and the sample type in humans.

Changes in bacterial composition due to beta diversity were determined using the Aitchison distance. Redundancy analysis (RDA) was performed controlling for mouse or human ID and adding covariates such as subsegments and groups (genotype or phenotype). The groups were collinear in the model adjusted by ID, and variance was not calculated for these variables. In mice, 12.35% (adjusted = 11.82%) of the variation of the microbial community was explained by sample types and subsegments (constrained), and the ID explained 53.68% of this variation (conditioned). The 33.97% of the variance was not explained by the model (unconstrained). Furthermore, the sample types and subsegments were both significant to the composition of the microbiota community evaluated with ANOVA analysis (F = 1.59, *p* = 0.033 and F = 16.88, *p* = 0.001, respectively) (Figure 3(f)).

For the human samples, we observed that 1.29% (adjusted = 1.36%) of the variation was explained by the sample type and the ID explained 92.34% of this variation, while 6.38% of the variation was not explained by the model. The sample types had a significant effect on the composition of the mucus-associated microbiota evaluated after ANOVA (F = 1.51, *p* = 0.018) (Figure 3g; Supplementary Figure 5a-b).

To further assess the similarity in the structure of the microbiota between the sample types, we compared the Aitchitson distances by paired sample types. The analysis was stratified by subsegment in mice, as the variance was significantly explained by this covariate in our previous analysis. In the ileum, the mucosal washes were more similar to the intestinal content compared to the other sample types. This suggests that the mucosal washes differ from the other low biomass samples in the ileum. In the cecum and colon, the intestinal content and the scraping seemed to be more similar compared to the other sample types. The use of mucosal washes or brushes leads to more similar microbial compositions than to the intestinal content and mucosal content (Figure 3(h)).

In humans, the use of mucosal washes and brushes leads to a significantly different microbial composition than the one obtained from the biopsy, suggesting a greater dissimilarity to the bacterial community evaluated with biopsies (Figure 3(i)).

**Figure 4.**
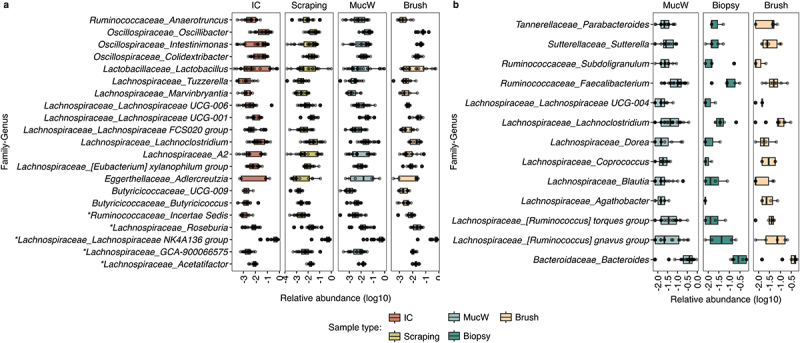
Evaluation of the relative abundance at genus level between sample types per host. Abundance was assessed between sample types in a) mice (left) and b) humans (right). The x-axis represents the relative abundance, and on the y-axis the genera present in the samples with a frequency greater than 4 per host. *p < 0.05 significant genera comparing the abundance between sample types grouped by subsegment significance evaluated with Dunn-test. IC: Intestinal content; MucW: Mucosal washes.

The evaluation of the taxonomy of all sample types was compositional in both hosts (Supplementary Figures S6 and S7). The most prevalent bacteria had similar abundances in the sample types (Figure 4(a–b)). However, to determine whether taxonomic differences were observed between sample types, we compared the relative abundance of bacteria at the phyla and genus taxonomic levels. In mice, we previously found an important influence of the subsegments on the diversity of the mucus-associated microbiota, so we made comparisons by stratifying with this covariable. When comparing at the phyla level, we observed high similarity between mucosal washes and scrapings, especially for the dominant Firmicutes and Bacteroidota, while differences were observed between brushes and intestinal contents in most subsegments (Supplementary Figure S8). At the genus level, we observed statistical differences between the intestinal content and the brushes, mucosal washes or scrapings in the cecum and colon and genus abundances were comparable between the sample types (Figure 4(a)). In human samples, we also observed no significant change in abundance between sample types at the phyla or genus level.

## Discussion

Numerous studies have highlighted the importance of assessing the mucus-associated microbiota in the host.^5^ However, comprehensive comparisons of sampling methods for this purpose are limited. Our results show that mucosal washes and brushings are a viable alternative to biopsies in humans or scraping in mice and provide a reliable profile of the mucus-associated microbiota. By focusing on mice, humans, and the primary preclinical and clinical settings, respectively, we evaluated samples commonly used for mucus-associated microbiota studies. Specifically, our study included mouse genotype as a variable to assess how mucosal washes perform under conditions that resemble human disease, such as IBD.^32^ This study does not aim to determine the precise extent of the effect of TLR5 deficiency or Crohn’s disease (CD) on microbiota differences, nor can this be separated from other confounding factors such as housing conditions in our current experimental design.

We used quantitative PCR to determine 16S rRNA gene copies per sample type.^33^ As expected, intestinal contents yielded higher bacterial loads than scrapings, mucosal wash and brush samples, particularly for mouse cecum and colon. This is consistent with previous reports showing higher bacterial cell counts in the colon than in the small intestine.^15^ Importantly, despite differences in total DNA extraction, the bacterial DNA yield from mucosal wash samples was comparable to scrapings and brushings in mice and even exceeded that from human biopsies.^12^ This supports previous findings that mucosal washes provide sufficient bacterial DNA for downstream analysis, even when the total DNA content varies.

Mucosal washes have previously been used for microbiota assessment, with suggestions that the resulting higher bacterial DNA and lower contamination from host DNA content may be of benefit for low-biomass samples.^10,12,15^ Our study shows that mucosal washes and brushes had lower host DNA content compared to scraping samples in mice or biopsies in humans, as assessed by relative quantification of 16S rRNA and 18S rRNA, particularly in the cecum or colon subsegments. Our findings confirm that most of the DNA in mucosal washes and brushes are of bacterial origin.

We evaluated whether the amount of host DNA affected sequencing performance by comparing total reads, mitochondrial-assigned reads, and bacterial reads. In mice, mitochondrial reads were similar across mucosal washes, brushes, and scrapings. However, this pattern differed in human samples, where mucosal washes consistently yielded higher bacterial reads compared to biopsies and brushings. This observation is consistent with previous reports of lower bacterial yields from human biopsies, which tend to have higher mitochondrial and chimeric reads.^12^ Although our results differ in some respects from previous studies, the variation within each host and sample type highlights the importance of considering host DNA when assessing mucus-associated microbiota.

Similar to previous studies showing higher richness estimates (Chao1) and Shannon diversity in the lumen than in the mucosa of mice,^15,34^ we observed lower alpha diversity in the mucosal washes than the luminal content. When comparing mucosal washes with scrapings and brushings, the results were less consistent: scrapings showed lower Shannon diversity, while brushings showed higher evenness. In humans, mucosal washes had greater diversity and evenness than biopsies, with brushings differing only in Shannon index. These findings support previous observations that biopsies often yield lower microbial biomass than aspirates or lavages, likely due to differences in sample collection.^10,11^

We found that for beta diversity in mouse samples, the sample type and subsegment significantly influenced the overall composition of bacterial communities, with subsegment being the main factor in combination with high intra-individual variation. As previously reported in mice from intestinal content samples,^35^ the sample-type groups emerged as significant predictors of beta diversity in humans. Our results are consistent with previous work evaluating human duodenal aspirates and biopsies^36^ and sigmoid colon lavage, brushing and biopsy samples,^12^ where sample type was one of the main factors explaining variance in the microbiota community.

When collecting mouse mucosa or brush samples, we observed that either of these methods, rather than scraping, had a minimal effect on the overall composition of the bacterial communities. This suggests that the choice between mucosal washes or brushes and scraping does not significantly affect the estimated microbial composition of mouse samples. In humans, mucosal washes and brushes were also closer to each other compared to biopsies. This shows that mucosal washes or brushes could provide similar information for microbiota analysis in both human and mouse hosts.

Brush samples have been used previously to assess the lumen or mucus microbiota in patients.^37,38^ It has been shown that the assessment of epithelial-associated microbiota using brushes was comparable to that observed on biopsies in the ileal pouch,^39^ which in our study seemed to be closer to the mucosal washings. Interestingly, it has been reported that lavages could be more contaminated or enriched on bacteria compared to samples such as biopsies or brushes.^12^ In contrast, our analysis throughout the mitochondrial assessment and evaluation of diversity does not fully support the previous.

In our study, Firmicutes and Bacteroidota predominated among mucus-associated bacteria in both host models, with Proteobacteria also prevalent in human ileal biopsies and mucosal wash samples. These results are consistent with previous studies of colon biopsies where similar taxonomic compositions were observed, independently from the use of amplicon-based or shotgun sequencing approaches.^14^ Overall, mucosal washes were compositionally similar to the other types tested, and only minor differences were observed at the genus level between the intestinal content and the brushes on cecum and colon in mice.

Although our results in humans showed that the mucosal wash and brush samples recovered a more significant proportion of bacterial reads, the taxonomy of the mucus-associated microbiota from the terminal ileum was not strongly influenced by the choice of sampling method. Other studies have evaluated the microbiota between biopsies and mucosal samples by brushing the intestinal epithelial.^13,39^ And like other studies,^39^ we detected that the microbial taxonomy detected in biopsies and brushes was the same. This supports the feasibility of using ileal mucosal washes or brushes as a reproducible surrogate for mucosal biopsies.^11^ Some of the advantages of brushes could be their use for cytology^40^ and histology,^41–43^ to evaluate further questions on the intestine. However, studying the microbiota and further experiments from the same sample of a brush could be difficult without the help of other sample types to complement the information from the results.

The mucus microbiota has been studied along and across the intestine^44,45^ where the sample type could be used based on the exploration of a dependent research question. As mentioned above, biopsies and brushings have been used to study bacteria that could be in close proximity to the intestinal epithelium due to the way the samples are collected. As shown by our results, the microbial communities assessed using lavages or washes represent those inhabitants of the mucus layer situated between the lumen, here represented by the intestinal content, and the intestinal mucosa, represented by the biopsies. Overall, these results provide a comprehensive overview of the microbial communities in the intestine and in closer contact with the host tissue.

Differences in extraction protocols could affect the outcome of microbiota assessment.^17^ Here, we compared biopsies extracted with a bead-beading method and the remaining sample types, which were extracted with an enzymatic method. We showed a significantly higher difference in the initial DNA extracted but lower total bacterial DNA. The beta diversity differed between these samples but not at the taxonomic level. Bead beating is part of the mechanical lysis steps in nucleic acid extraction for various samples, such as tissue or stool, due to their high cellular or bacterial biomass content, respectively. Therefore, in biopsies, bead beating facilitates tissue dispersion. However, for the low-biomass samples compared in this study, including mucosal washes, brushes, and scrapings, the bead beating may exacerbate DNA degradation, as suggested by previous reports.^46,47^

In contrast to our result, another study in oral mouthwash, a low-biomass sample type, showed lower levels of total DNA from samples extracted by mechanical bead-beating compared to those extracted by enzymatic lysis, but they did not observe differences in beta or alpha diversity per method.^46^ While studies reporting on wastewater DNA extraction found higher bacterial DNA for gram-positive bacteria compared to gram-negative bacteria for bead-beating, and a higher result for both bacteria when extraction was combined with proteinase K and bead-beating, which may enhance the detection of difficult-to-lyse bacterial cells.^48,49^ Although we acknowledge that interpretation of our results on less common bacteria should be made with caution, the most common bacteria did not differ in their abundance between human sample types, and the biological interpretations based on them are robust.

The use of amplicon-based amplification could be a limitation in detecting species or strains, as well as specific differences at such granular levels between sample types. The integrated analysis of different host-derived readouts, such as histopathology or immunology, together with the microbiome and other omics from the same region in animal models and humans may be limited if they are all based on the same sample type. Nevertheless, our results demonstrate the comparability of mucosal washes from different experimental conditions in animal models and humans to assess the mucus-associated microbiota.

## Conclusions

In conclusion, our comprehensive assessment of mucus-associated microbiota in both mice and humans provided compelling evidence for the suitability of mucosal wash samples or brushes as alternatives and complementary sample types to traditional biopsies or other tissue-derived samples such as scrapings. This study confirms that the microbial composition recovered from these methods is comparable, while also highlighting the advantage of mucosal washes in their ability to sample different regions of the intestine, particularly in mice. Additionally, we observed a close similarity between mucosal washes and brushes, thereby providing further support for these methods as reproducible techniques in the field of mucus-associated microbiota research.

## Supplementary Material

Supplemental Material

## Data deposition

BioProject ID: PRJNA1043131
