# Supplementary material for: Mucosal washes are useful for sampling intestinal mucus-associated microbiota despite low biomass
Source: Gut Microbes. 2025 Feb 20;17(1):2464296. doi: 10.1080/19490976.2025.2464296 (PMC11849919; doi:10.1080/19490976.2025.2464296)
Supplement: Supplemental Material [file KGMI_A_2464296_SM6672.zip › Supplementary_material_R2.docx]

**Mucosal washes are useful for sampling intestinal mucus-associated microbiota despite low biomass**

**Martinez-Medina, Jennifer N^a,b,c^, Ghazisaeedi, Fereshteh^d,e^, Kramer, Catharina^a,f^, Ziegler, Jörn F^a,f,g^, McParland, Victoria^a,b,c^, Mönch, Paul W^a,f^, Siegmund, Britta^a,f^, Jarquín-Díaz, Víctor Hugo^a,b,c*#^, Fulde, Marcus^d,e*^, Forslund-Startceva, Sofia K^a,b,c,g,h,i*^**

^a^Charité – Universitätsmedizin Berlin, corporate member of Freie Universität Berlin and Humboldt-Universität zu Berlin.

^b^Max Delbrück Center for Molecular Medicine (MDC), Berlin, Germany.

^c^Experimental and Clinical Research Center, a Cooperation of Charité-Universitätsmedizin and the Max-Delbrück Center, Berlin, Germany.

^d^Institute of Microbiology and Epizootics, School of Veterinary Medicine at the Freie Universität Berlin, Robert-von-Ostertag-Str. 7, 14163 Berlin, Germany.

^e^Veterinary Centre for Resistance Research (TZR), School of Veterinary Medicine at the Freie Universität Berlin, Robert-von-Ostertag-Str. 8, 14163 Berlin, Germany.

^f^Department of Gastroenterology, Infectious Diseases and Rheumatology, 12203 Berlin, Germany.

^g^Berlin Institute of Health at Charité – Universitätsmedizin Berlin, BIH Biomedical Innovation Academy, BIH Charité Junior Clinician Scientist Program, Charitéplatz 1, 10117 Berlin, Germany.

^h^DZHK (German Centre for Cardiovascular Research), Partner Site Berlin, Germany.

^i^Structural and Computational Biology, European Molecular Biology Laboratory, Heidelberg, Germany.

*These authors shared the supervision of the project.

**Corresponding author:**

**#Víctor Hugo Jarquín-Díaz** ([VictorHugo-DiazJarquin@mdc-berlin.de](mailto:VictorHugo-DiazJarquin@mdc-berlin.de))

**Affiliations:**

- Max Delbrück Center for Molecular Medicine (MDC), Berlin, Germany.
- Experimental and Clinical Research Center, a Cooperation of Charité-Universitätsmedizin and the Max-Delbrück Center, Berlin, Germany.

**ORCiD:** [**https://orcid.org/0000-0003-3758-1091**](https://orcid.org/0000-0003-3758-1091)


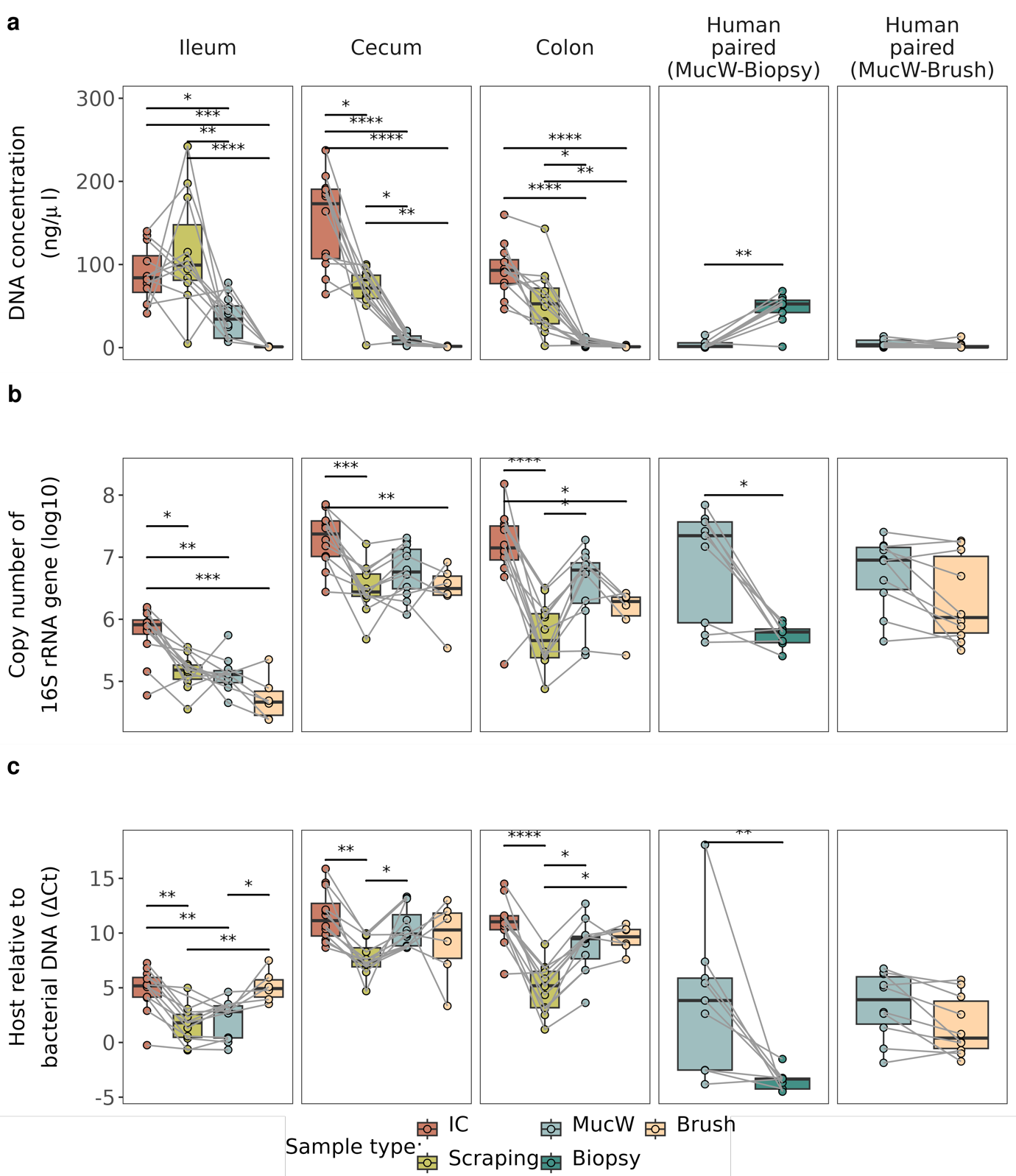


**Supplementary Figure 1:** **a)** DNA concentration, **b)** bacterial load (copy number of 16S rRNA gene in log10), and **c)** host relative to bacterial DNA (ΔCt) between samples within subsegments in mice ((n = 12 for intestinal content, mucosal washes and scrapings, and n = 6 for brushes per subsegment) and in the paired mucosal wash-biopsy human samples (n = 9), and paired mucosal wash-brush (n = 10). IC: intestinal content; MucW: Mucosal washes.

**Supplementary Figure 2: a-b)** Scatterplots with histogram representing the p-scores generated with the combined-either method based on frequency (x-axis) and prevalence (y-axis) using the Decontam library. Colours represent the ASVs classified as true (yellow) or false (blue) contaminants, and bars represent the distribution of the ASVs. **a)** mice samples, **b)** human samples. **c** and **e)** Lollipop graph representing the frequency based on the abundance of the ASVs classified as contaminants, y-axis names are a combination of the family and genus, and the colour of the point represents the phylum to which the ASV belongs, **c)** mice samples, **e)** human samples. **d** and **f)** Lollipop graph representing the prevalence of the ASVs classified as contaminants, y-axis names are a combination of the family and genus, and the colour of the point represents the phylum to which the ASV belongs. **d)** mouse samples, **f)** human samples.

**Supplementary Figure 3:** **a)** Total read counts, **b)** mitochondrial read counts, and **c)** filtered read counts represented in logarithm 10 between sample types within subsegments in mice (n = 12 for intestinal content, mucosal washes and scrapings, and n = 6 for brushes per subsegment), paired mucosal washes-biopsy human samples (n = 7 biopsies and n = 7 mucosal washes; two of the biopsy samples were discard during the filtering process for the analysis of the microbiota), and paired mucosal washes-brushes human samples (n = 10 mucosal washes and n = 10 brushes from same patients). IC: intestinal content; MucW: Mucosal washes.

**Supplementary Figure 4:** **Heatmap of the relative abundance of the phyla and genera in mice**. On the y-axis the genus and phyla and the x-axis each column represent one sample. Samples were grouped by sample types and subsegment.

**Supplementary Figure 5:** **Heatmap of the relative abundance of the phyla and genera in humans**. On the y-axis the genus and phyla and the x-axis each column represent one sample. Samples were grouped by sample types.

**Supplementary Figure 6:** **Comparison of the abundance of Firmicutes and Bacteroidota between the sample types within the different subsegments in mice**. X-axis (bottom) represents the sample types classification, x-axis (upper) classficiation per subsegment of the sample types, y-axis (left) represents the relative abundance, y-axis (right) classification of the Firmicutes or Bacteroidota. IC: intestinal content; MucW: Mucosal washes.
